# Small-Scale High-Pressure Hydrogen Storage Vessels: A Review

**DOI:** 10.3390/ma17030721

**Published:** 2024-02-02

**Authors:** Jian Li, Xingzai Chai, Yunpeng Gu, Pengyu Zhang, Xiao Yang, Yuhui Wen, Zhao Xu, Bowen Jiang, Jian Wang, Ge Jin, Xiangbiao Qiu, Ting Zhang

**Affiliations:** 1 Nanjing Institute of Future Energy System, Nanjing 211135, China; lijian@njiet.cn (J.L.); chaixingzai@njiet.cn (X.C.); guyunpeng@njiet.cn (Y.G.); zhangpengyu@iet.cn (P.Z.); yangxiao@iet.cn (X.Y.); 2 Institute of Engineering Thermophysics, Chinese Academy of Sciences, Beijing 100190, China; 3 School of Energy and Mechanical Engineering, Nanjing Normal University, Nanjing 210042, China; 221912002@njnu.edu.cn; 4 North Night Vision Science & Technology (Nanjing) Research Institute Co., Ltd., Nanjing 211135, China; xuzhao_nuaa@126.com (Z.X.); bwjiang1992@hotmail.com (B.J.); wangjian0106211@163.com (J.W.); jgtm@sina.com (G.J.); ndqxb@163.com (X.Q.); 5 Innovation Academy for Light-Duty Gas Turbine, Chinese Academy of Sciences, Beijing 100190, China; 6 University of Chinese Academy of Sciences, Nanjing 211135, China

**Keywords:** hydrogen storage, high-pressure vessels, fiber composite winding, liner materials, glass capillary

## Abstract

Nowadays, high-pressure hydrogen storage is the most commercially used technology owing to its high hydrogen purity, rapid charging/discharging of hydrogen, and low-cost manufacturing. Despite numerous reviews on hydrogen storage technologies, there is a relative scarcity of comprehensive examinations specifically focused on high-pressure gaseous hydrogen storage and its associated materials. This article systematically presents the manufacturing processes and materials used for a variety of high-pressure hydrogen storage containers, including metal cylinders, carbon fiber composite cylinders, and emerging glass material-based hydrogen storage containers. Furthermore, it introduces the relevant principles and theoretical studies, showcasing their advantages and disadvantages compared to conventional high-pressure hydrogen storage containers. Finally, this article provides an outlook on the future development of high-pressure hydrogen storage containers.

## 1. Introduction

It has become a widespread consensus of the international community to build a “decarbonized” society and mitigate climate disasters, such as global warming and tsunamis, caused by traditional fossil fuels [1,2]. Therefore, countries have been seeking more environmentally friendly, more easily accessible, and efficient green energy for energy transition. The current renewable green energies are mainly inclusive of wind, solar, tidal, biomass, and hydrogen energy [3,4,5,6]. As an ideal source of clean energy, hydrogen energy is completely clean and green because its combustion product with oxygen is only water. Moreover, hydrogen is the most abundant element on earth and the energy source with the highest energy density per unit mass. As shown in Table 1, the energy released by hydrogen can be as high as 142 MJ/kg, which is 3.25 times that of gasoline and 3.4 times of that of natural gas. The conversion efficiency of hydrogen to work in fuel cells is about 60%, while the conversion efficiencies of fossil fuels in motors, such as gasoline and diesel, are only 22% and 45% [7,8], respectively. This further confirms the potential of hydrogen as a suitable alternative energy source.

In the hydrogen energy system, hydrogen production serves as the foundation, while hydrogen storage and transportation are prerequisites for large-scale applications. Presently, hydrogen production technology has reached a high level of maturity [9,10]. However, due to the small size of hydrogen molecules, they can penetrate even the most common tank materials, leading to significant hydrogen losses. Additionally, the flammability of hydrogen (particularly when it comes into contact with air) and the safety concerns surrounding its storage and transportation present enormous challenges for the development of hydrogen storage technology, consequently limiting the potential scenarios where hydrogen energy can be utilized. As such, the primary objective in the utilization of hydrogen energy is to pursue safe, efficient, cost-effective, and more energy-efficient hydrogen storage technologies [11]. At present, high-pressure gaseous hydrogen storage [12], cryogenic liquid hydrogen storage [13], solid-state hydrogen storage [14], and organic liquid hydrogen storage [15] represent the main methods for storing hydrogen through compression, liquefaction, physical absorption, and chemical binding. Compared to the other options, high-pressure gaseous hydrogen storage boasts several advantages, including a simple equipment structure, low energy consumption during compressed hydrogen preparation, fast charging and discharging of hydrogen, and a good adaptability to a wide range of temperatures. This method is currently the most commonly used and mature hydrogen storage technology, and it is expected to remain dominant for an extended period in the future [16]. The U.S. Department of Energy (DOE) has set a goal for the hydrogen mass density of domestic on-board hydrogen batteries to reach 4.5 wt% by 2020, 5.5 wt% by 2025, and ultimately 6.5 wt% [17]. This establishes the benchmark for all high-pressure hydrogen storage vessels.

This review presents an overview of the current state of high-pressure hydrogen storage vessels. While there have been numerous reviews on hydrogen storage, most have focused on technologies such as metal hydride hydrogen storage and liquid hydrogen storage [18,19,20]. This paper aims to specifically report on high-pressure hydrogen storage technologies, including various innovative high-pressure hydrogen storage vessel variants and preparation processes, such as capillary hydrogen storage and microsphere hydrogen storage, among others. Additionally, various hydrogen storage vessel mechanisms and their respective advantages and disadvantages are presented in detail, along with recent developments in high-pressure hydrogen storage technology and the remaining challenges that need to be addressed.

## 2. Traditional High-Pressure Hydrogen Storage Vessels

High-pressure hydrogen storage vessels are a key technology for the widespread use of compressed hydrogen, which is widely used in hydrogen refueling stations and on-board hydrogen storage [21]. Almost 80% of hydrogenation processes over the world utilize the high-pressure storage vessel in both hydrogen storage and transportation fields [22]. To satisfy the industrial requirement of the hydrogen storage density, the internal pressure should be increased up to 70 MPa [23]. Both gravimetric and volumetric hydrogen storage densities will increase by raising the hydrogen pressure. However, the emergence of safety issues and high manufacturing costs are the main obstacles against its commercialization [24].

Currently, high-pressure hydrogen storage vessels have gradually evolved from all-metal vessels (type I vessels) to non-metallic liner fiber fully wound vessels (type IV vessels) [25]. Several types of high-pressure hydrogen storage vessels are shown in Table 2.

### 2.1. Type I and II Vessels

The development of type I vessels (metal pressure vessels) was driven by the industrial needs of the late 19th century, particularly the storage of carbon dioxide used to produce carbonated beverages. As early as 1880, wrought iron vessels were reported to be used for hydrogen storage and for military purposes, with storage pressures up to 12 MPa. The storage pressure of type I vessel was greatly enhanced in the late 1880s with the invention of pressure vessels made of seamless steel tubes manufactured by drawing and forming in England and Germany. By the 1960s, the working pressure of type I vessels had increased from 15 MPa to 20 MPa [26], as shown in Table 2. In these type II vessels, the metallic wall is wrapped with a fiber resin composite on the cylindrical part [27]. Compared to type I, they have 30–40% less weight at the expense of a 50% higher cost [28]. However, classic metal pressure vessels are not capable of developing high strength/stiffness to weight ratios, so the application scenario is limited.

Due to the molecular permeation of hydrogen, steel vessels are easily corroded by hydrogen, and thus suffer from hydrogen embrittlement, leading to vessel failure under high pressure and risks such as bursting [29]. They are generally used for stationary, small capacity hydrogen storage. In recent years, the research on type I and II vessels have mainly focused on the seamless processing of metals and the failure mechanism of metal gas vessels. In particular, different test methods have been used to evaluate the fracture toughness properties of metallic materials in gaseous hydrogen [30]. Generally, a rough selection of materials can be made via disc pressure testing, environmental hydrogen tensile tests, or fracture toughness and crack extension tests, which can be used to detailly examine the proportional decrease in the parameters, such as the section shrinkage, yield strength, and critical stress strength factor, by hydrogen. ISO/TR15916:2004 [31] The Hydrogen System Safety Fundamental Issues provided hydrogen embrittlement sensitivity data for some materials for reference, as shown in Table 3 [31].

### 2.2. Fiber Composite Winding Gas Vessel

**Figure 1:**
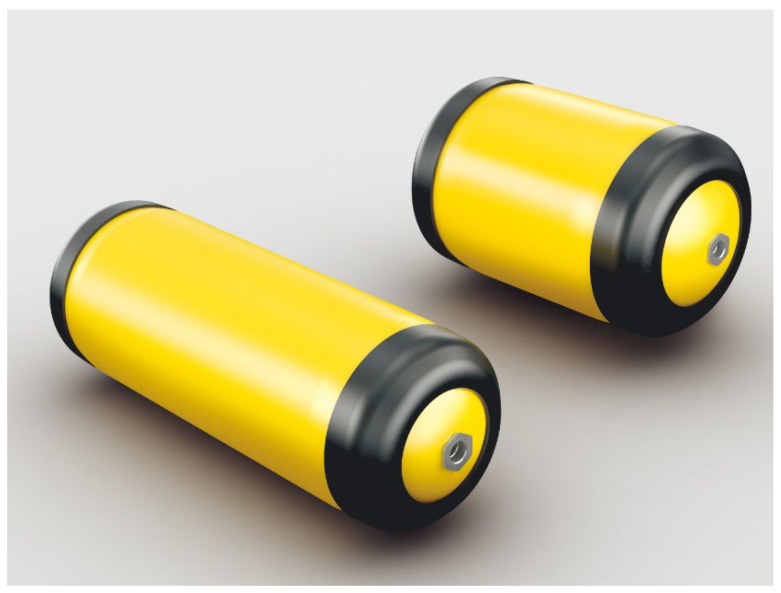
Toyota MIRAI’s three-layer structure of a high-pressure vehicle-mounted hydrogen storage vessel; reproduced from https://global.toyota/en/newsroom/toyota/22740159.html (accessed on 25 January 2024) Copyright Toyota.

At a pressure of 1 bar, the density of hydrogen is 0.1 g/L, and the energy volumetric density is 0.0033 kWh/L. When the pressure increases to 700 bar, the density and energy volumetric density become 40 g/L and 1.32 kWh/L, respectively. As hydrogen pressure increases, both its gravimetric and volumetric hydrogen storage densities also increase. Classic metal pressure vessels have a relatively low strength/stiffness to weight ratio. Composite filament winding technology has the potential to enhance the storage performance due to its lightweight, high-strength, and appropriate resistance for both fatigue and corrosion [32,33]. The gravimetric capacity of steel vessels is less than 1/4 that of composite pressure vessels operating at the same pressure [34]. Type III and type IV vessels are the mainstream vessels manufactured by fiber composite winding. Type III vessels have an aluminum liner and type IV vessels have a polymer liner. The fiber composite material is wound around the periphery of the liner in the form of spirals and hoops to increase the structural strength of the liner. The liner acts as a barrier between the hydrogen and the composite layer to prevent hydrogen from escaping through microcracks in the composite layer substrate [35]. The best representative in the field of vehicles is the Japanese Toyota Mirai Type IV hydrogen storage vessel with a plastic liner and fiber winding (Figure 1), which has a rated working pressure of 70 MPa, a high gravimetric hydrogen storage density of 5.7 wt%, and a volumetric hydrogen storage density of 40.8 g/L [36].

With these features, composite filament wound technology will be suitable for aerospace, military, hydrospace, and vehicular applications. Thus, the rapid commercialization of hydrogen energy in the automotive sector will be observed [37].

#### 2.2.1. Preparation Process of Filament-Wound Gas Vessels

**Figure 2:**
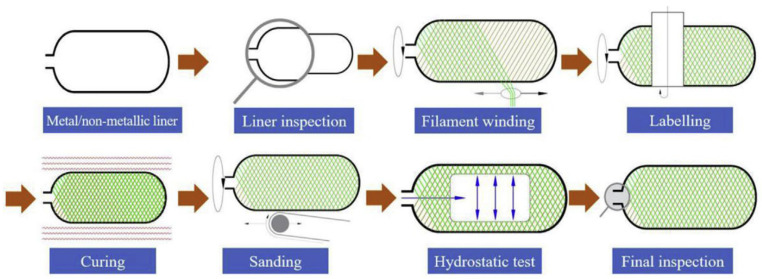
The composite vessel manufacturing process. Reprinted from Ref. [38] Dongliang Wang 2019, used with permission. Copyright Elsevier.

A composite vessel is fabricated from a liner over-wrapped with continuous filament windings, as shown in Figure 2 [38]. The first step is to make the thermoplastic into an inner liner and then check the surface for wrinkles, dents, and other defects. The second step is to enter the fiber winding process, affix a label after curing, and then polish the outside surface. The final step is to conduct the final product test after passing the hydrostatic test. This review focuses on the inner liner molding process and filament winding process.

##### Molding Process of the Liners

The type IV hydrogen vessel liner molding process involves injection and welding molding, blow molding, and rotational molding. After considering the advantages and disadvantages of various molding processes. The process of injection and welding is more developed in comparison [39]. The end of the plastic liner head is molded using injection molding. During the molding process, the processed metal boss is placed in the injection mold cavity. Then, the melted liner material is injected into the cavity through the injector to form the body of the metal boss and plastic head. Finally, the product is obtained through cooling. The middle vessel part of the plastic liner is created using extrusion molding. Extrusion molding involves the extrusion of molten materials through a mold with a specific interface shape, followed by cooling to obtain a straight vessel product [40].

Extrusion blow molding is typically used to blow molding plastic liners. The molten tubular material is extruded by the extruder and then wrapped by the blow mold, and then blown to the middle of the melt to make the melt close to the inner wall of the mold. The plastic liner is then cooled and formed [41].

Rotary molding involves adding powder or liquid materials to the mold, rotating it around two mutually perpendicular axes, employing even heat on the mold, and then cooling it to obtain the liner. Rotational molding can achieve both the integral molding of the head and vessel, as well as the connection of the metal boss and plastic [42]. However, controlling the uniformity of the wall thickness and material density is challenging due to the pressureless molding process and technical difficulties.

The fabrication process of the liner has an impact on its gas barrier properties, molding accuracy, dimensional stability, and interfacial bond strength. The different processes have their own advantages and disadvantages (Table 4). In order to accelerate the development of type IV hydrogen storage vessels, molding standards for each process should be developed and the relationship between the process parameters and lining performance should be revealed. In addition, the demand for large, complex, precise, and efficient liner molding technology is equally urgent.

##### Molding Process of Filament Winding

Both the middle layer and outer layer of hydrogen storage vessels are made using the filament winding process. The fiber winding process includes wet winding, dry winding, and semi-dry winding. Wet winding is used to impregnate the fiber and wind it directly onto the liner. Dry winding is used to heat the prepreg on the winding machine to a viscous flow state and wind it onto the liner. Semi-dry winding is used to impregnate the fiber, then pre-dry it and wind it onto the liner [43,44]. The advantages and disadvantages of the different processes are shown in Table 5.

The main study on the fiber winding process currently lies in the use of finite element simulation to optimize the winding method and winding angle. Factors such as the winding angle of the composite layer, thickness of winding layer, and pressure distribution with the inner liner have a great influence on the performance of composite gas vessels [47,48,49]. How to choose the best design parameters to make the relevant materials reach the maximum performance is the key issue of composite gas vessel design. Some scholars have measured the law of the winding angle affecting gas vessel performance through experiments and obtained some practical conclusions. Onder A [50] investigated the effect of the operating temperature and fiber winding angle on the ultimate strength of composite gas vessels using experimental and finite element methods, and finally obtained the optimal design structure for this type of vessel. Hopmann et al. [39]. found that the winding angle had a great influence on the tensile and compressive strength of the structure by conducting experiments on three glass fiber epoxy resin tubes with different winding angles. Mertiny P et al. [51] concluded that a proper winding angle can greatly improve the structural performance by conducting performance experiments on a large number of different fiber tubes.

However, how to form an effective connection between the optimized design of the winding angle and the actual performance is the research idea that is beneficial to the industry so that the design concept can be put into engineering practice. Krikanov A [52] used the optimal design method based on the lattice theory to find the winding angle at the minimum vessel mass with the composite vessel mass as the objective function and the strength parameter as the constraint function. Based on the classical laminate theory, Rosenow et al. [53] explored the variation law of stress and strain on the lining and winding layers of a composite material pressure vessel from 15° to 85° of the winding angle and concluded that the optimum winding angle for balanced winding vessels is 55°. Based on the three-dimensional composite failure theory, Parnas L et al. [54] used a theoretical approach to optimize the winding angle of composite gas vessels and analyzed the effect of the winding angle on the ultimate strength of the vessels.

Another aspect of the research on winding technology is the arrangement and thickness of the fiber wound layers. Adali et al. [55] used the Tsai–Wu strength criterion and elasticity theory to predict the maximum value of the internal pressure that a composite pressure vessel could bear and optimized the lay-up sequence of the composite using the Ruben multidimensional method. Messager T et al. [56] investigated the effect of the lay-up sequence of the fibers on the composite load-bearing properties of cylindrical shells, provided the optimal lay-up of the fibers, and performed numerical simulations and experimental verifications of the cylindrical shells with wound layers made from both glass fiber/epoxy and carbon fiber/epoxy materials. David et al. [57] took the angle and thickness of the wound layer as the design variables and used the simplified conjugate gradient method based on the genetic principle to select the optimal winding angle and thickness of the fiber layer while minimizing the maximum value of the Mises stress of the lining.

#### 2.2.2. Material of Type IV Hydrogen Storage Vessels

**Figure 3:**
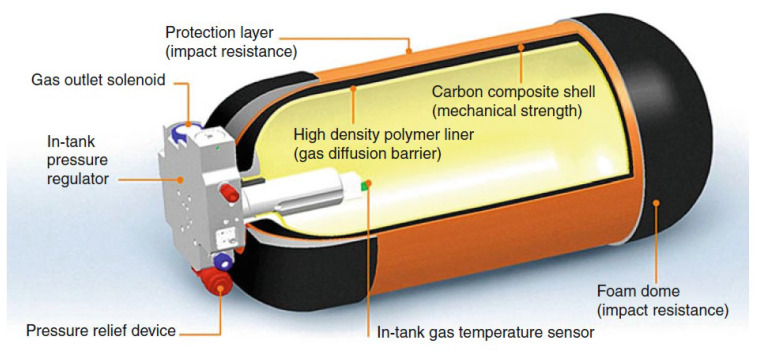
Quantum Technologies Type IV compressed gaseous hydrogen storage vessel. Ref. [58] Von Helmolt R 2007, used with permission. Copyright Elsevier.

Type IV hydrogen storage vessels are made of non-metallic materials except for the metal vessel valve seat. Taking the Quantum Technologies Type IV vessel as an example, the internal structure of the type IV hydrogen storage vessel consists of the following parts, as shown in Figure 3 [58].

(i) The total thickness of the vessel wall is about 20~30 mm.
(ii) Innermost gas barrier layer: In direct contact with hydrogen, with a thickness of about 2~3 mm, it is an olefin plastic polymer, which plays the role of the hydrogen barrier.
(iii) Middle pressure-resistant layer: Carbon fiber-reinforced composite material (carbon fiber and epoxy resin) with the thickest layer, and the thickness of this layer is minimized to improve the hydrogen storage efficiency under the premise of ensuring the pressure-resistant grade.
(iv) Outermost protective layer: Glass fiber-reinforced composite material (glass fiber and epoxy resin) with a thickness of about 2~3 mm.

Type IV hydrogen vessel have several failure modes, the first of which is hydrogen permeation [59]. The second mode of failure is thermal (high and low temperature) instability and thermal aging, resulting from exposure to extreme temperatures and temperature changes. This can cause a reduction in the mechanical properties and hydrogen barrier properties [60,61]. The third type of failure is caused by stress and strain [62,63]. The failure modes mentioned above necessitate the materials that exhibit a good resistance to permeability, stability to high and low temperatures, and high strength.

##### Liner Materials

The matrix materials commonly used for type IV hydrogen vessel liners include high-density polyethylene (HDPE), polyamide (PA), polyethylene glycol terephthalate (PET), and other polyether-based materials [64]. The common matrix needs to be modified for the combined characteristics, such as the barrier properties, aging resistance, strength, and thermal stability to address the complex failure factors and maximize the benefits of lightweighting [28]. Optimizing the materials can be achieved through strategies such as filling the functional materials, optimizing the aggregated state, and enhancing functionalization by designing process parameters or employing novel methods.

**Figure 4:**
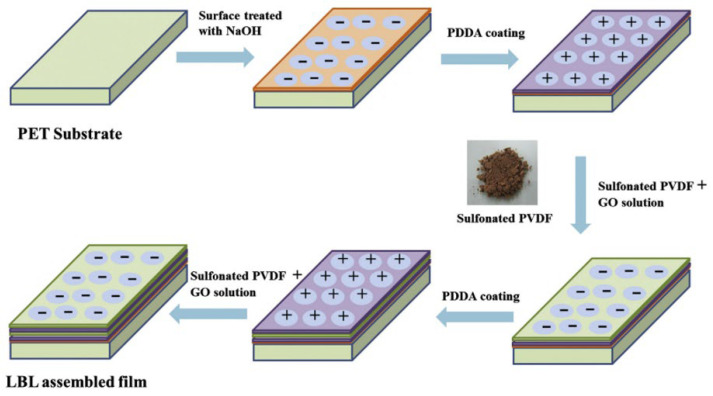
Schematic for the preparation of the LBL assembly. “−” for negative charge; “+” for positive charge Ref. [65] Rajasekar R 2013, used with permission. Copyright Elsevier.

Rathanasamy et al. [65] fabricated multi-layered films containing poly(diallyldimethylammonium) chloride (PDDA) and sulfonated polyvinylidene fluoride (SPVDF)–graphene oxide (GO) composites through layer-by-layer (LBL) assembly to enhance the hydrogen gas barrier properties, as shown in Figure 4. To determine the impact of modified organoclay on the barrier properties of HDPE, María C. C et al. [66] conducted a study on the permeation of CO_2_ in films of high-density polyethylene (HDPE) and organoclay modified with polyvinylalcohol (MMTHDTMA/PVA). The results indicated a significant improvement in the barrier properties due to the incorporation of modified organoclay. Incorporating a load of 2 wt% of MMTHDTMA/PVA into the polymer matrix resulted in a 43.7% decrease in CO_2_ flow compared to pure polyethylene. The barrier properties of composites are affected by fillers with varying properties, contents, aspect ratios (or specific surface areas), and even different microstructures of the same filler [67,68].

##### Carbon Fiber-Reinforced Polymer/Plastic (CFRP)

Glass fibers, silicon carbide fibers, alumina fibers, boron fibers, carbon fibers, and aramid and Poly-p-phenylene benzobisoxazole (PBO) fibers are used in the manufacturing of fiber composite wound vessels, of which carbon fibers are gradually becoming the mainstream fiber material due to their excellent performance (such as Toray’s T300, T700, T1000) [69]. Table 6 lists several common mechanical properties of fibers.

Composite material gas vessels made of high-strength and high-modulus carbon fiber materials through winding technology not only have a reasonable structure and light weight, but also have a wide application space for the preparation of hydrogen storage vessels with good technology and designability. The carbon fiber bundle consists of 10,000~50,000 carbon fibers with an average diameter of 5 to 8 μm. The weight ratio of the epoxy resin layer to carbon fiber layer is 20–30:70–80 [70]. Between the skeleton carbon material and the adhesive, there is not only physical adhesion but also chemical bonding, and the aromatic epoxy resin provides higher strength than the aliphatic, such as bisphenol A epoxy resin. However, the high viscosity will make the impregnation operation difficult, and it is difficult to impregnate the interior uniformly [71]. Therefore, the viscosity during impregnation should be adjusted with epoxy resins or solvents with low polymerization. The disadvantage is that the impregnation must be repeated to regulate the large fluctuations of volume shrinkage.

Therefore, research on the development and modification of resin system has mainly focused on the mechanical properties, thermal stability, and process properties. Cha et al. [72] functionalized CNT by adding melamine to improve the dispersion of CNT in the epoxy resin matrix and enhance the interfacial bonding between CNT and the matrix. It was found that the fracture toughness of the epoxy resin increased by 95%, Young’s modulus increased by 64%, and the tensile strength increased by 22% when M-CNT was added at a 2% mass fraction. Lee et al. [73] used polyethersulfone (PES)-modified epoxy resin and found that the tensile strength, impact strength, fracture toughness, and thermal stability of the obtained PES/epoxy composites were increased by 44%, 35%, 11%, and 1%, respectively.

## 3. Novel High-Pressure Glass Hydrogen Storage Vessels

Oxidic glasses are considered promising candidates for cost-effective, safe, and long-term storage of pressurized hydrogen in microvessels, such as hollow microspheres and capillaries. This is because metals are prone to material embrittlement and cracking well below the initial yield stress, and polymers cannot offer low enough permeation rates [74]. The greatest advantage of glass over steel is its higher strength and lower weight. As a result, high pressure resistance can be achieved with a small wall thickness and less material. Hollow glass microspheres [75] and glass capillary arrays [76] for hydrogen storage vessels have received increasing attention in recent years.

### 3.1. Hollow Glass Microspheres (HGM)

**Figure 5:**
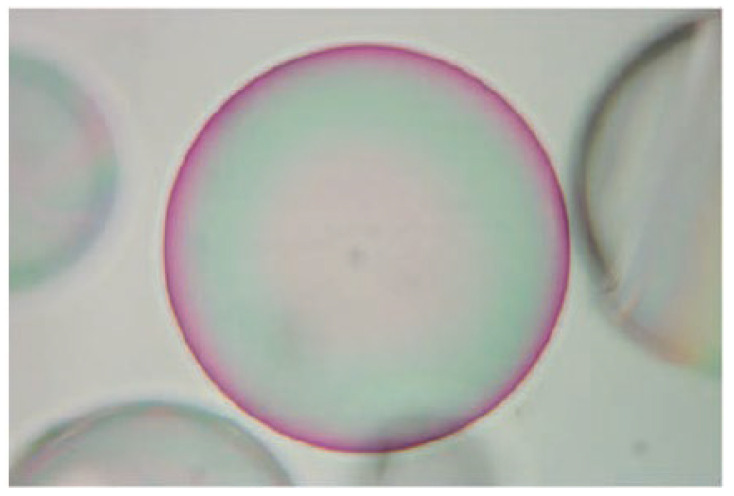
Photo of a hollow glass microsphere. Ref. [75] Kohli D K 2008, used with permission. Copyright Elsevier.

Hollow glass microspheres (HGMs) are a novel category of materials that have an interconnected porous network on the microsphere wall. The photograph of the system is shown in Figure 5 [75]. The HGM particle size is normally between 1 and 100 mm, although the diameter sizes range from 5 mm to 100 mm, wall thickness from 2~3 mm, and the nanopores of the microsphere walls from 100 to 500 nm [77,78,79]. It has the advantages of non-toxic, self-lubrication, good dispersion and fluidity, high pressure resistance, low thermal conductivity, heat preservation, fire resistance, etc. As early as 1977, Teitel [80] proposed the use of micron-sized HGMs as a high-pressure hydrogen storage vessel and made a series of studies on it. The results showed that the hydrogen storage mass density of a HGM could reach the target value calibrated by the U.S. DOE vehicle hydrogen storage vessel in that year, and it was a very promising high-pressure hydrogen storage vessel.

#### 3.1.1. Hydrogen Charging and Discharging Mechanism of HGMs

The hydrogen charging and discharging of HGMs is mainly achieved by penetrating the glass walls of the microspheres [81]. At low temperature or room temperature, the hollow glass microspheres show non-permeability. When the temperature increases to the range of 300–400 °C, the penetration rate of the HGM gradually increases, allowing hydrogen to enter into the microsphere under a certain pressure (10–200 MPa). At this moment, when the temperature is lowered to room temperature, the penetration of the glass body gradually decreases again, and the hydrogen gas remains inside the hollow glass microsphere, which means that the hydrogen gas is stored. The temperature is raised again to realize the release of hydrogen. The whole HGM is a physical process, and the system is not affected by impurities (compared to metal hydride). As a hydrogen storage vessel, the HGM requires fast diffusion of hydrogen when charging and discharging hydrogen, while slow diffusion of hydrogen is desired when storing hydrogen. The diffusion coefficient can be expressed as a relation of the Arhenius formula [82].

(1) $$K=K_{0}exp(-\frac{E}{RT})$$

where K is the gas diffusion rate (mol/m·S·Pa), K_0_ is the diffusion constant (mol/m·S·Pa·K), E is the diffusion activation energy (J/mol), R is the gas constant (8.31 J/mol·K), T is the thermodynamic temperature, and *K*_0_ and *E* are determined by the gas type and the amount of the substance of the network outer body (adjusting body) in the glass. For silicate glasses, the molar fraction of the network outer body is denoted by *M.* Then, Equation (2) can be written as the following [83].

(2) $$K_{0}\approx \left[3.4+\left(8\times 10^{-4}\right)M^{3}\right]\times 10^{-17}$$

E≈3600+165M

Hydrogen charging and discharging can be achieved by controlling the atmospheric conditions and the temperature of the environment in which the hollow glass microspheres are placed. When *P*_0_ > *P_i_*, hydrogen gas penetrates inside the sphere; when *P_i_* > *P*_0_, hydrogen gas penetrates outside the sphere. *P_i_* and P_0_ denote the air pressure inside and outside the microsphere, respectively. If permeation is desired, the ambient temperature will increase; if permeation is not desired (in the case of storage, transportation, etc.), the temperature will decrease.

#### 3.1.2. Hydrogen Storage Efficiency of HGMs

**Figure 6:**
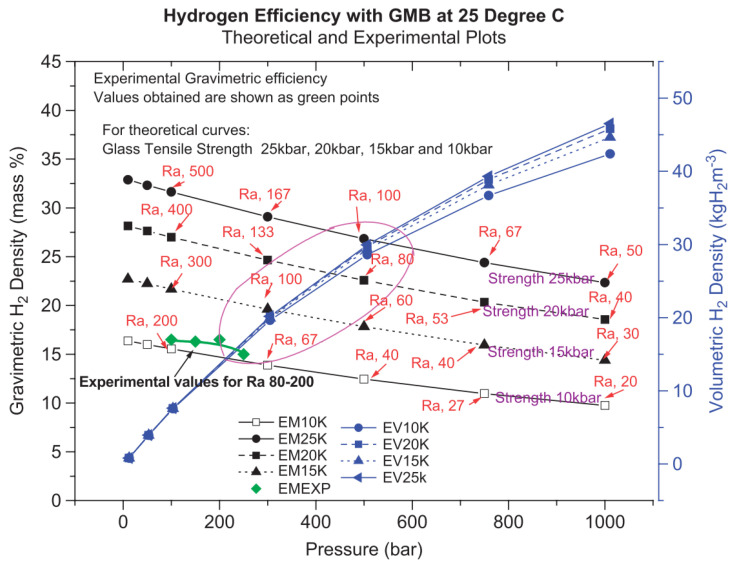
The variation of the gravimetric and volumetric efficiencies of HGMs with pressures for the different glass strengths. Ref. [75] Kohli D K 2008, used with permission. Copyright Elsevier.

The rate of hydrogen transfer depends on the hydrogen pressure across the microsphere walls, temperature, glass composition, and microsphere size. Kohli et al. investigated the effect of the HGM glass strength on the hydrogen storage efficiency [75]. The results showed that a higher glass strength allowed the vessel to withstand high pressures in a thinner shell, thus increasing the weight and volume efficiency of hydrogen storage. The weight hydrogen storage efficiency of the HGM reached 15–25% at a hydrogen storage pressure of 50 MPa. The effect of the ratio between the radius and wall thickness of the HGM on the hydrogen storage efficiency was also investigated. The ratio of the radius to wall thickness was considered to be the best when it was between 80 and 120. When the ratio of radius to wall thickness was 100, the weight hydrogen storage efficiency of HGM with glass strength of 2.5 GPa reached 27% at 50 MPa. In contrast, the weight hydrogen storage density of the HGM with a glass strength of 1.5 GPa reached 20% at a pressure of 30 MPa. Even in the medium pressure range, the HGM with a high aspect ratio had a good hydrogen storage performance. Figure 6 shows the variation of the weight and volume efficiency of the HGM with pressures for the different glass strengths [75].

Considering that hydrogen can only diffuse into the interior and exterior of a HGM through the microsphere walls, accelerated diffusion often requires an elevated temperature. However, at high temperatures, the hydrogen pressure inside the microsphere may exceed the breakage limit of the microsphere. In addition, due to the insufficient thermal conductivity of the glass, a lot of heating is required during the hydrogen charging and discharging process. Meanwhile the shape and size of HGMs make the hydrogen release rate low. All these hinder the further application and development of HGM. Research has showed that the release rate of stored hydrogen molecules can be improved by properly doping HGMs using metal dopants (for example, magnesium and iron [84], cobalt [85,86], zinc [87], etc.). Dalai et al. concluded that the heat transfer of the HGM improved with the addition of Mg and Fe loading, resulting in an increase in the hydrogen storage capacity of the final product. The adsorption of hydrogen on the HGM loaded with Mg and Fe began at a temperature of 200 °C and a pressure of 10 bar.

**Figure 7:**
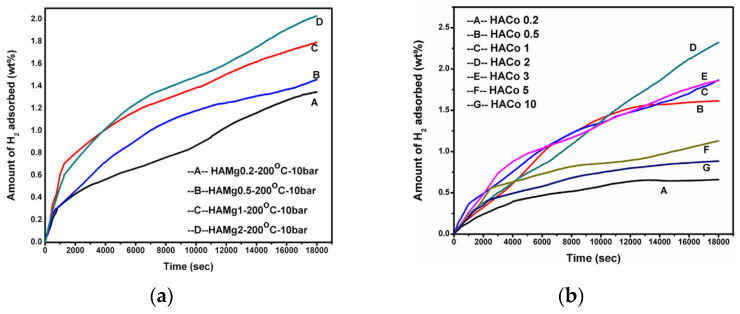
(**a**) Hydrogen uptake curve for HAMg 0.2, 0.5, 1, and 2 at 200 °C. Ref. [84] Dalai S 2014, used with permission. Copyright Elsevier. (**b**) Hydrogen uptake curve for HACo 0.2, 0.5, 1, 2, 3, 5 and 10 at 200 °C. Ref. [85] Dalai S 2017, used with permission. Copyright Elsevier.

The hydrogen uptake on magnesium and cobalt-loaded HGMs are shown in Figure 7. The hydrogen gas uptake on the HGMs increased with an increase in the magnesium concentration up to 2 wt% but decreased with further increases to 3 wt%. Beyond 2%, nanocrystals of MgO and Mg formed on the HGMs, leading to pore closure and a reduction in the hydrogen storage capacity [84]. This conclusion was also reached for cobalt-loaded HGMs [85,86].

**Figure 8:**
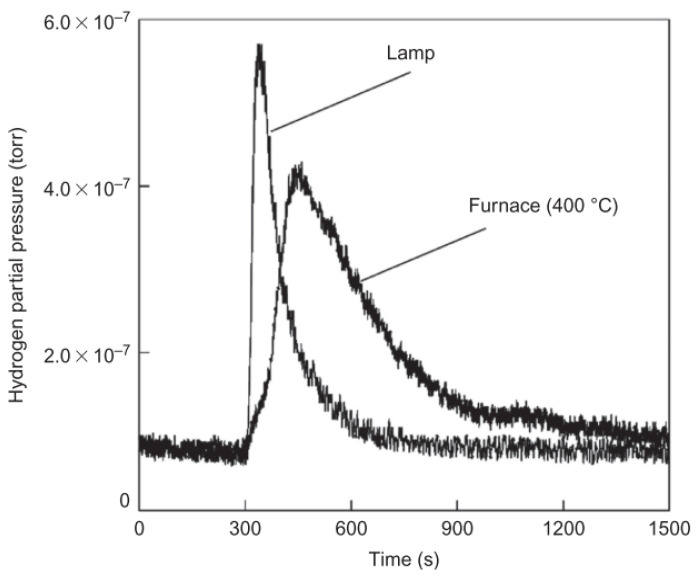
Effect of the heating method on the hydrogen outgassing response in 0.5 wt% Fe_3_O_4_-doped Corning Glass Works commercial glass 7070. Ref. [88] Zhevago N K 2016, used with permission. Copyright Elsevier.

Whereas an HGM is a thermal insulator, fast heating and cooling of many spheres are, at best, difficult and this method is not applicable for most commercial applications. Shelby and colleagues [88] applied various types of radiation (e.g., microwave, infrared, ultraviolet, ultrasonic, etc.) to the HGM with the hope of enhancing and controlling the permeation of hydrogen through the glass. It was found that infrared radiation exhibited a significant effect. In particular, it was observed that hydrogen permeation through borosilicate glass plates doped with specific metal oxides (F_3_O_4_, CoO, NiO, V_2_O_5_, or Cr_2_O_3_) was accelerated when exposed to incandescent lamps compared to heating. These HGM samples were successfully filled to 10.3 MPa and 34.5 MPa with minimal loss due to the fragmentation of the microspheres, and the hydrogen release rates obtained by selectively exposing the metal oxide doped glasses to lamp radiation were superior to those obtained from the furnace (Figure 8). Compared to furnace heating, undoped metal oxide glasses exhibited a poor hydrogen release response. When doped metal oxide glasses exposed to lamp illumination, the hydrogen release increased exponentially with the applied voltage (i.e., IR radiation intensity) and the hydrogen release rate increased with the metal oxide and hydrogen oxide concentration.

The contribution of infrared radiation to increase the hydrogen-retrieving rate was unclear. Indeed, the transmission loss in the doped glass prevented infrared radiation from penetrating at a sufficient depth into the bulk. Microwave radiation with longer wavelengths could be more effective as it can penetrate deeper. For this to work, the glass dopants must have a high absorption coefficient in the microwave radiation frequency range. Fullerene C60 and other carbonaceous materials are suitable candidates [89].

### 3.2. Glass Capillary Arrays

To solve the problems of hydrogen embrittlement in high-pressure vessels and the inefficiency of charging and discharging hollow glass microspheres, Professor N.K. Zhevago et al. proposed the use of glass capillary arrays to store hydrogen [90]. Each single fiber acts as an independent pressure vessel. The thousands of single fibers then form multi-fiber, thin hollow glass fibers for the storage of gases. A large number of multi-fibers are combined to create complex structures that are free in shape and volume, depending on the application and desired amount of stored hydrogen. One major drawback of conventional high-pressure storage systems is their dependence on shape for optimal force distribution. For instance, the edges experience a peak of high structural stress, which can cause the structure to break if the stress is too high. To prevent these pressure peaks, common vessels have a cylindrical shape.

**Figure 9:**
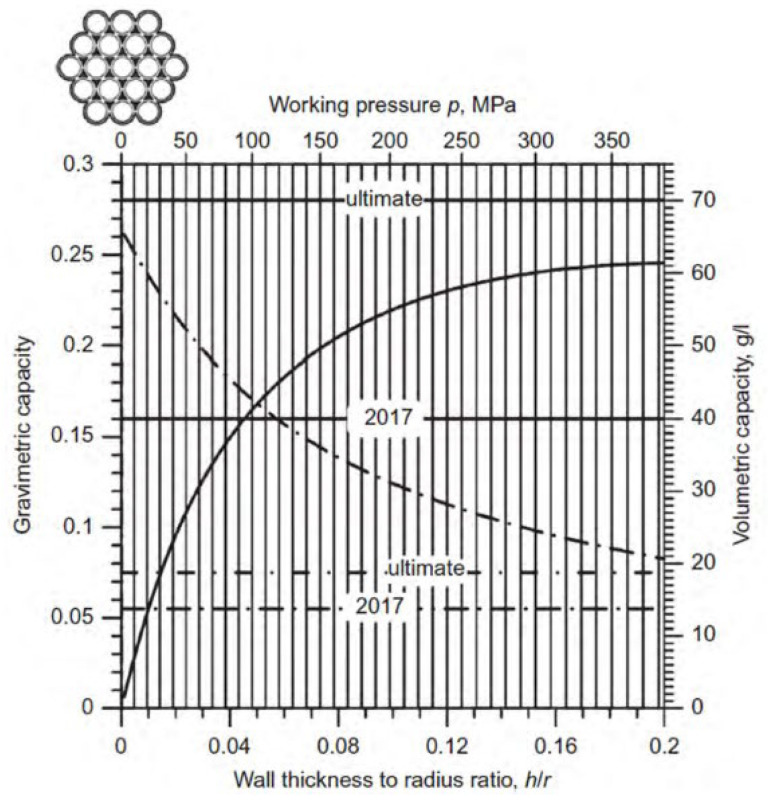
Volumetric (solid curves and right *y*-axis) and gravimetric capacity (dash-dotted, left) of the S-2 glass capillary array at room temperature versus the ratio of the capillary wall thickness to the radius (or the working hydrogen pressure). The DOE target values are shown by the corresponding horizontal lines. Ref. [91] Zhevago N K 2016, used with permission. Copyright Elsevier.

According to theoretical calculations (Figure 9) [91], the hydrogen storage density per unit mass can be greater than 7 wt% when the ratio of the capillary wall thickness to the radius is less than 0.2, and the hydrogen storage density per unit volume can be greater than 30 g/L when the working pressure is greater than 70 MPa. These indexes are already comparable to the highest level of the Japanese Toyota Mirai IV hydrogen storage vessel. This provides the opportunity to safely store and transport hydrogen under high pressure and can be used in all types of fuel cell systems used in the vehicle. The strength and safety of the glass capillary array for hydrogen storage is similar to that of the HGM, but the amount of hydrogen in each capillary is very small compared to standard high-pressure storage vessels, thus preventing the possibility of explosion due to mishandling or accidents. The capillary array has an ideal form and size compared to a storage vessel. Compared with HGMs, capillary arrays have more space for use. The charging and discharging of hydrogen in a glass capillary can be done by means of plugs or regulating valves instead of regulating the hydrogen diffusion by temperature [92].

#### 3.2.1. The Mode of Glass Capillary Array Charging and Discharging

Hydrogen can be stored in glass capillaries using various filling and closing procedures, depending on the application or the planned storage period. In both cases, the capillaries are sealed at one end by melting. The glass capillary array is also made of glass, and therefore can be filled and released in the same way as the HGM. That is, hydrogen charging and discharging is achieved by adjusting the temperature based on the permeation theory [81]. However, this requires closing the ends of the glass capillary array by melting.

Another method is to use a special low-melting alloy for sealing the capillary tubes. First, individual arrayed capillary glass tubes are placed in special containers, which are evacuated and filled with hydrogen to the storage pressure, and then the system is heated to the melting temperature of the alloy. At this point, the metal is pressed into the individual or arrayed capillary tubes using special equipment. After cooling, the alloy solidifies, and the hydrogen is sealed in the glass tubes. In the practical environment, the alloy is simply heated to the melting point and the hydrogen is released as the metal is squeezed by the high pressure of the hydrogen inside. The amount of pressure released can be controlled by the heating rate [92].

To avoid the need for heating energy to improve the efficiency in order to penetrate the alloy or plug alloy, it is feasible to connect the capillary tube to the microvalve [93]. By connecting the amount of glass with a capillary structure to an adapter with a special microvalve, storage systems suitable for short-term storage and/or an alternating supply of hydrogen can be prepared. These microvalves are electromagnetically driven with a very short switching time and are connected to a pressure sensor that ensures the required pressure and flow rate by means a section of pre-volume. Moreover, this control allows for in situ filling without affecting the hydrogen output. The process starts by connecting the microvalves to a hydrogen refueling station, which is filled with hydrogen after the pressure in the vessel has been evacuated. When the storage pressure is reached, the microvalve closes. Then, the system is disconnected from the filling station and is ready for use.

#### 3.2.2. Study on the Pressure Resistance of Glass Capillary Hydrogen Storage Vessels

**Figure 10:**
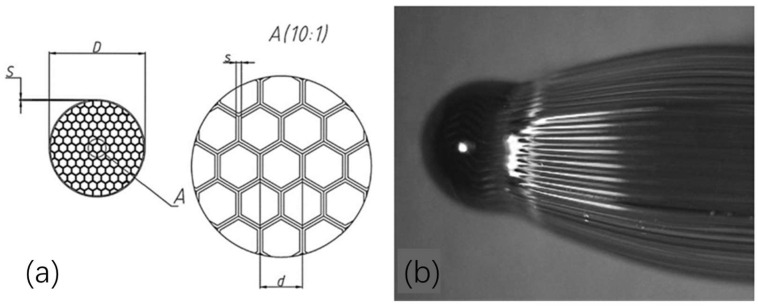
(**a**) Schematic view of the cross-section of the honeycomb capillary array from the borosilicate glass used in the experiments. (**b**) Photo of a sealed tip of the glass capillary array. Ref. [94] Zhevago N K 2010, used with permission. Copyright Elsevier.

Zhevago et al. [94], researchers from the Russian National Research Center “Kurchatov Institute”, conducted the first experiment of encapsulating hydrogen gas into capillary arrays. This experiment tested two types of glass capillaries. The first capillary was made of quartz with an outer diameter of nearly 480 μm and a wall thickness of 25 μm. They were sealed at one end and covered with a thin layer of epoxy resin, which was fused together by an epoxy polymerization reaction via UV radiation. Another capillary array was made of borosilicate glass. Tightly stacked hexahedral capillaries ware placed inside cylindrical glass cladding and merged together during placement. The geometry of the array is shown in Figure 10. The hydrogen encapsulation was accomplished using a metal stopper inside the autoclave.

The experimental results showed that the highest-pressure values were obtained using the epoxy-reinforced quartz capillary arrays. They withstood up to 171 MPa pressure of hydrogen stored at room temperature, resulting in a 48.3 g/L volumetric and 10.25% gravimetric capacity of the storage medium. The corresponding results for the borosilicate honeycomb arrays were lower, i.e., 15.6 g/L volumetric and 3.2% gravimetric.

Tests by the German Federal Institute also determined the pressure resistance of single and multiple (array) capillaries (Table 7) [91]. These tests were related to the capillary glass materials (quartz, borosilicate, aluminosilicate, and soda-lime glass), capillary size, wall thickness, and durability. These tests were performed to determine the optimal parameters and materials for the use of glass capillaries in various applications. The results showed that borosilicate capillaries were capable of withstanding pressures in excess of 100 MPa. Durability testing further confirmed that borosilicate capillaries consistently outperformed the other glass materials over thousands of refill cycles, with the highest average value (100.2 MPa) and the highest rupture pressure rate (124.2 MPa)

An important determinant of the pressure resistance of glass capillaries is related to defects in the glass structure, such as air bubbles, grooves, or crack defects. The theoretical strength of glass is about 70 GPa, while the measured tensile strength is usually much lower [95]. This large discrepancy is due to the presence of small defects in the glass, especially on the surfaces subjected to tensile stresses that can become stress concentrators, and under certain conditions, cracks initiated by defects can continue to expand [96]. Ronald Meyer-Scherf [97] of the University of Magdeburg, supported by the project “Characterization of glass capillaries for gas storage under high pressure” of C.En.co, analyzed various series of hollow glass capillaries for defects and distinguished between defects in the material itself and production-related defects at the source. Aging due to environmental influences and their effect on the compressive strength of the internal loads were also examined. The results showed that material-related defects were caused by the irregular network structure of the glass material Their appearance was influenced by the chemical composition and the number of additives. The relevant defects were compared to the production-related defects, which had a negligible effect on the compressive resistance. Production-related defects were the parameter that had the greatest impact on the compressive resistance. Such defects can occur both as volume defects and surface defects. In particular, defects on the surface can produce significant stress peaks under applied loads. To reduce such defects, it can be achieved, for example, by applying a coating to the surface.

In addition, the hydrogen tightness of high-pressure hydrogen storage is a basic criterion for long-term storage. MARC et al. [98] performed a geometric optimization of a glass capillary by determining the hydrogen permeation coefficient of epoxy resin and glass lacquer. It was demonstrated that the hydrogen permeability coefficient of epoxy resins was slightly higher than that of borosilicate glass, and that the hydrogen permeability coefficient of epoxy resins did not change significantly with curing conditions. The data suggests that 700 bar of hydrogen will only halve after 2.5 years in borosilicate glass arrays. Consequently, the capillary glass tube/epoxy encapsulation system has promising applications in mobile high-pressure hydrogen storage vessels.

## 4. Conclusions and Outlook

As countries around the world pay attention to the development and utilization of hydrogen energy, hydrogen storage will certainly become key to the hydrogen energy utilization industry. High-pressure gaseous hydrogen storage, as the only commercial hydrogen storage technology, has been developed significantly since 1970. Among them, high-pressure hydrogen storage vessels are continuously developing towards light-weight, high-pressure, and high-gravimetric/volume hydrogen storage densities. At the same time, with the upgrading of composite fiber materials, polymer materials, winding equipment, and winding technology, high-pressure hydrogen storage vessels will certainly expand their applications. However, while the vessel performance is improving, further research on the hydrogen embrittlement phenomenon and the failure mechanism of high-pressure hydrogen storage vessels are needed to standardize the production and testing of vessels to continuously improve the safety performance of high-pressure hydrogen storage vessels. At the same time, it is necessary to reduce the manufacturing cost of high-pressure hydrogen storage vessels.

Secondly, it is obvious that glass hydrogen storage vessels are a promising hydrogen storage technology, which achieves the requirements of safety, high efficiency, light weight, and high pressure for hydrogen storage, and the absence of hydrogen embrittlement is a major advantage of glass hydrogen storage vessels. Especially, capillary array hydrogen storage vessels can customize their size and shape, and they are expected to be used as energy storage devices for various portable devices. At present, the mechanism of glass hydrogen storage vessels is clear, but the processing technology and supporting valve-type devices are not mature. Therefore, one of the main directions of future works is to improve these aspects.

## Figures and Tables

**Table 1:** Properties of hydrogen, gasoline vapor, and natural gas [7,8].

Technical Index	Hydrogen	Gasoline Vapor	Natural Gas
Explosion limit (%)	4.1~75	1.4~7.6	5.3~15
Burning point energy (MJ)	0.02	0.2	0.29
Diffusion coefficient in air (m^2^·s^−1^)	6.11 × 10^−5^	0.55 × 10^−5^	1.61 × 10^−5^
Energy density (MJ·kg^−1^)	143	44	42

**Table 2:** Different types of hydrogen storage vessels [25].

Type	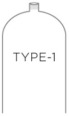	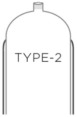	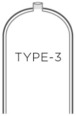	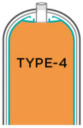
Material	All steel	Fiberglass hoop wrap, steel liner	All-carbon full wrap, metallic liner	Fiberglass/carbon full wrap, plastic liner
Working pressure (MPa)	17.5–20	26.3–30	30–70	>70
Media compatibility	Hydrogen brittle, corrosive	Hydrogen brittle, corrosive	Hydrogen brittle, corrosive	Hydrogen brittle, corrosive
Mass hydrogen storage density (wt%)	≈1	≈1.5	≈2.4–4.1	≈2.5–5.7
Volumetric hydrogen storage density (g/L)	14.28–17.23	14.28–17.23	35–40	38–40
Service life (years)	15	15	15–20	15–20
Cost	Low	Moderate	Highest	High
Is the car available?	No	No	Yes	Yes

**Table 3:** Hydrogen embrittlement sensitivity data for some commonly used metals [31].

Metal		Extreme Hydrogen Embrittlement	Serious Hydrogen Embrittlement	Light Hydrogen Embrittlement	Negligible Hydrogen Embrittlement
Aluminum	1100				✔
	6061-T6				✔
	7075-T73				✔
Be-Cu25				✔	
Cu					✔
Ni270			✔		
Alloy steel 4140		✔			
Carbon steel	1020		✔		
	1042 (normalized)		✔		
	1042 (quenching and tempering)	✔			
Maraging steel, 18Ni-250		✔			
Stainless	A286				✔
	17-7PH	✔			
	304 ELC			✔	
	305			✔	
	310				✔
	316				✔
	410	✔			
	440C	✔			
	Nickel alloys 718	✔			
Ti and titanium alloys	Ti			✔	
	Ti-5Al-2.5SN (ELI)		✔		
	Ti-6Al-4V (annealing)		✔		
	Ti-6Al-4V (STA)		✔		

✔ represents the degree of hydrogen embrittlement of the metal.

**Table 4:** Comparison of the advantages and disadvantages of the liner molding process [40,41,42].

Molding Process	Injection Molding and Welding Molding	Blow Molding	Rotational Molding
Advantages	Dimensional stability Low cost Free design of sealing structures Thin-walled products can be prepared High impact toughness High resistance to environmental cracking	Uniform wall thickness Simple forming process Low tooling cost Large parts can be molded	High production efficiency High impact toughness High resistance to environmental cracking
Disadvantages	Welding required Many work processes Difficult to form large-sized structures	Poor dimensional stability Low denseness; easy to form defects High requirements for the melt flow rate High energy consumption for batch production Low production efficiency	Low tooling cost Uneven wall thickness High requirements for the melt flow rate Special structure with inserts and large-sized structures are difficult to mold The surface of the sealing part generally requires subsequent processing.

**Table 5:** Comparison of the advantages and disadvantages of the filament winding process [45,46].

Winding Process	Wet Winding	Dry Winding	Semi-Dry Wingding
Advantages	Lower cost than dry windingGood parallelism of fiber alignment Less fiber wear during wet winding High production efficiency	Good quality of products Clean winding machine Good labor hygiene High product quality	It is easy to operate without prepreg molding process. Good product quality; low bubble content in resin.
Disadvantages	Resin waste and poor operating environment It is not easy to control the glue content and quality of the finished products Less variety of resin available for wet winding	The winding equipment is expensive and requires additional prepreg yarn manufacturing equipment, so the investment is larger. The interlayer shear strength of dry winding products is low.	Larger investment in winding equipment Poor process adaptability

**Table 6:** Fiber mechanical properties.

High Performance Fibers	Modulus of Elasticity (GPa)	Tensile Strength (MPa)	Elongation (%)
E-Glass Fiber	74	3510	1.38
S-Glass Fiber	84	4920	1.97
Aramid fiber	121	3790	2.61
T300 Carbon Fiber	230	3530	2.0
T700 Carbon Fiber	235	4900	2.74
T1000 Carbon Fiber	304	6330	3.54

**Table 7:** Burst pressure measured for various capillaries [91].

Glass Composition	Density (g/cm^3^)	Outside Diameter (μm)	Inside Diameter (μm)	Length (mm)	*P_u_*^max^ (MPa)	*P_u_*^min^ (MPa)
Soda-lime	2.52	400	300	100	114.7	25.0
Borosilicate	2.33	400	360	200	124.2	73.7
Aluminosilicate	2.65	340	300	200	62.7	32.6
Quartz	2.2	400	300	200	109.1	39.4
